# Impact of COVID-19 pandemic on pharmacologic treatment of patients newly diagnosed with osteoporosis

**DOI:** 10.1371/journal.pone.0291472

**Published:** 2023-09-13

**Authors:** Micaela White, Lauren Hisatomi, Alex Villegas, Dagoberto Pina, Alec Garfinkel, Garima Agrawal, Nisha Punatar, Barton L. Wise, Polly Teng, Hai Le

**Affiliations:** 1 Department of Orthopaedic Surgery, University of California Davis, Sacramento, CA, United States of America; 2 California Northstate University, College of Medicine, Elk Grove, CA, United States of America; 3 Department of Internal Medicine, University of California Davis, Sacramento, CA, United States of America; University of Campania Luigi Vanvitelli: Universita degli Studi della Campania Luigi Vanvitelli, ITALY

## Abstract

**Purpose:**

This study determined whether initiation of pharmacologic treatment was delayed for newly diagnosed osteoporosis patients during the COVID-19 pandemic.

**Methods:**

1,189 patients ≥50 years with newly diagnosed osteoporosis using dual-energy x-ray absorptiometry (DXA) screening at a single academic institution were included. Patients with previous osteoporosis were excluded. Patients diagnosed between March 1, 2018—January 31, 2020 (pre-pandemic cohort, n = 576) were compared to those diagnosed between March 1, 2020—January 31, 2022 (pandemic cohort, n = 613). Age, sex, race, ethnicity, ordering providers (primary vs specialty), and pharmacological agents were evaluated. Primary outcomes included proportion of patients prescribed therapy within 3 and 6-months of diagnosis, and mean time from diagnosis to treatment initiation.

**Results:**

The pre-pandemic cohort had more White patients (74.3 vs 68.4%, p = .02) and no differences between remaining demographic variables. Only 40.5% of newly diagnosed patients initiated pharmacologic therapy within 6 months. Patients treated at 3-months (31.8 vs 35.4%, p = 0.19) and 6-months (37.8 vs 42.9, p = 0.08) were comparable between cohorts (47.2 vs 50.2% p = 0.30). Mean time from diagnosis to treatment initiation was similar (46 vs 45 days, p = 0.72). There were no treatment differences based on gender, race, or ethnicity or between ordering providers (65.1 vs 57.4% primary care, p = 0.08). Bisphosphonates were most often prescribed in both cohorts (89% vs 82.1%).

**Conclusions:**

This is the first study assessing COVID-19’s impact on pharmacologic treatment of newly diagnosed osteoporosis. 40.5% of newly diagnosed patients were treated pharmacologically within six months of diagnosis, and the pandemic did not significantly affect treatment rates.

## Introduction

Osteoporosis is a major public health burden and a systemic skeletal disorder characterized by decreased bone mineral density (BMD) and deterioration of bone architecture [1]. In the United States, approximately 10 million people over the age of 50 have osteoporosis, with an estimated economic burden of $17.9 billion annually [2, 3]. Compromised bone strength increases the risk of fragility fractures, which reduce quality of life while increasing mortality [4, 5]. Consequently, for patients newly diagnosed with osteoporosis, timely pharmacologic treatment is essential to prevent fragility fracture and reduce mortality [1].

Despite remarkable advances in the diagnosis and management of osteoporosis, a care gap still exists as many patients with osteoporosis are left untreated or undertreated [6]. Between 2008 to 2012, the use of oral bisphosphonates in the United States declined by more than 50% due to patients’ safety concerns about the medications [7]. More recently, Zarowitz et al. reported only one-third of nursing home residents with osteoporosis were receiving pharmacologic therapy [8]. Even among patients with fragility fractures such as hip or vertebral compression fractures, osteoporosis treatment rates were alarmingly low [9–13]. The undertreatment of osteoporosis, coupled with today’s aging population, presents a challenge for current and future healthcare providers as the number of fragility fractures continues to rise [14].

To make matters worse, the COVID-19 pandemic has presented considerable challenges in the care of patients with chronic diseases, including osteoporosis. In particular, Fuggle et al. performed a global survey of osteoporosis healthcare professionals in 2021 and observed an increase in telemedicine appointments, delay in dual-energy x-ray absorptiometry (DXA) scanning, and limited availability of osteoporosis medications for patients, thus further widening the osteoporosis care gap [15]. Moreover, a narrative review performed by Moretti et al. described the nutritional challenges people faced during the COVID-19 pandemic and their consequences on bone health. These challenges included a decrease in fresh food availability and increased alcohol consumption, overall food intake, and consequently increased body weight. Notably, this review found people were consuming foods with decreased set-up time, i.e. canned or frozen foods. In addition, this review reported a decrease in Vitamin D due to changes in diet and lack of sunlight [16]. As osteoporosis is an already undertreated chronic disease and public health burden, it is important that patients continue to receive appropriate osteoporosis care, even during a global pandemic [17].

It is important to note the above mentioned study by Fuggle et al. was a survey study [15]. To date, there has been no study directly measuring the impact of COVID-19 pandemic on the pharmacologic treatment of patients who were newly diagnosed with osteoporosis. In this study, we determined whether initiation of pharmacologic treatment was delayed for patients newly diagnosed with osteoporosis during the pandemic. We hypothesized the treatment rate was lowered and time from diagnosis to treatment delayed as a result of the pandemic.

## Methods

Institutional Review Board (IRB) approval from the University of California, Davis, School of Medicine was obtained for this study, and consent was waived by the IRB. This is a retrospective cohort study using review of electronic medical records (EMRs), and all data were fully anonymized before we accessed them for analysis. We evaluated all patients ≥50 years who underwent DXA scanning at a single academic tertiary referral center between March 1, 2018 to January 31, 2022. Each patient’s electronic medical record was reviewed for prior history of osteoporosis by assessing past medical visits, “osteoporosis” ICD diagnoses, previous DXA scans showing low BMD, health records from previous institutions, and self-reported history. If any of these parameters were present in the patient’s chart prior to the study dates, those patients were excluded. The review process of the medical records occurred between March 10, 2022 and May 25, 2022 and was performed by five medical students for the purposes of this research study. Only patients with newly diagnosed osteoporosis were included. This study determined a new diagnosis of osteoporosis based on the World Health Organization criteria of bone mineral density T-score of -2.5 or lower on DXA scan. Patients with osteoporosis diagnosed between March 1, 2018 to January 31, 2020 (pre-pandemic cohort) were compared to patients diagnosed between March 1, 2020 to January 31, 2022 (pandemic cohort). Basic demographics including age, sex, race, and ethnicity were evaluated. Sex was categorized dichotomously as male or female. Ethnicity was categorized into Hispanic, Non-Hispanic, and Unknown groups. Race was categorized into White, Asian, African American, Other, and Unknown groups. Primary outcomes included the proportion of patients who were initiated on pharmacologic therapy at 3-months and 6-months of diagnosis defined at the time of DXA scan, as well as the mean time from osteoporosis diagnosis to initiation of pharmacologic treatment. Ordering providers (primary care vs specialty care providers) and types of pharmacologic agents were also compared. Pharmacologic agents were grouped into bisphosphonate, denosumab, parathyroid hormone analogue (e.g., teriparatide, abaloparatide), or estrogen agonist/antagonist groups. Chi square tests were performed for categorical data, while independent t-tests were performed for continuous data, with significance set at 0.05.

## Results

During the study period, 11,335 DXA studies were performed, and we identified 1,189 patients who were newly diagnosed with osteoporosis. There were 576 patients in the pre-pandemic cohort and 613 in the pandemic cohort (Table 1). There was no significant difference between cohorts with regard to age (69.3 vs 68.8 years, p = 0.33), sex (87.0 vs 86.1% female, p = 0.67), or ethnicity (88.7 vs 86.9% Non-Hispanic, p = 0.35). However, there was a higher proportion of White patients in the pre-pandemic cohort (74.3 vs 68.4%, p = 0.02).

**Table 1:** Demographics of cohorts.

	Pre-Pandemic	Pandemic	P-Value
**Patients**	576	613	
**Age (years)**	69.3	68.8	0.33
**Female (n, %)**	501 (87.0%)	528 (86.1%)	0.67
**Ethnicity (n, %)**			
Non-Hispanic	511 (88.7%)	533 (86.9%)	0.35
Hispanic	56 (9.7%)	54 (8.8%)	
Unknown	9 (1.6%)	26 (4.2%)	
**Race (n, %)**			
White	428 (74.3%)	419 (68.4%)	0.02*
Asian	59 (10.2%)	71 (11.6%)	
African American	14 (2.4%)	20 (3.3%)	
Other	61 (10.6%)	72 (11.7%)	
Unknown	14 (2.4%)	31 (5.1%)	

Overall, only 40.5% of patients (n = 481) newly diagnosed with osteoporosis were started on pharmacologic therapy within 6 months of diagnosis. Proportions of patients treated at 3-months (31.8 vs 35.4%, p = 0.19) and at 6-months (37.8 vs 42.9, p = 0.08) were comparable between the pre-pandemic and pandemic cohorts (47.2 vs 50.2% p = 0.30) (Table 2). The mean time from osteoporosis diagnosis to initiation of pharmacologic treatment was similar (46 vs 45 days, p = 0.72). The ordering providers did not differ between cohorts (65.1 vs 57.4% primary care providers, p = 0.08). Bisphosphonates were the most often prescribed in the pre-pandemic (89%) and pandemic cohorts (82.1%) (Table 3).

**Table 2:** Treatment rates, time to treatment, and prescriber pattern.

	Pre-Pandemic	Pandemic	P-Value
**Patients**	576	613	
**3-Month Treatment (n, %)**	183 (31.8%)	217 (35.4%)	0.19
**6-Month Treatment (n, %)**	218 (37.8%)	263 (42.9%)	0.08
**Time to Treatment (days)**	46	45	0.72
**Primary Care Prescriber (n, %)** ^**a**^	142 (65.1%)	151 (57.4%)	0.08

^a^Of the patients who received treatment by 6-month, the proportion of patients who were prescribed by their primary care providers.

**Table 3:** Pharmacologic treatment by medication group.

	Pre-Pandemic	Pandemic
**Patients on Treatment**	218	263
**Bisphosphonate**	194 (89%)	216 (82.1%)
**Denosumab**	14 (6.4%)	26 (9.9%)
**Parathyroid Hormone Analogue**	7 (3.2%)	13 (4.9%)
**Estrogen Agonist / Antagonist**	3 (1.4%)	8 (3%)

In evaluating the entire cohort, there were no differences in medical treatment rates based on gender, race, or ethnicity. Female patients were treated 40.1%, and male patients were treated 42.5% (p = 0.56). White patients were treated 40.0%, and non-White patients were treated 42.4% (p = 0.47). Non-Hispanic patients were treated 40.7%, and Hispanic patients were treated 39.1% (p = 0.74) (Table 4).

**Table 4:** Comparison of overall osteoporosis treatment by gender, race, and ethnicity.

Variable	Female	Male	P-Value
Patients	1029	160	
Patients on medications	413	68	
Proportion on medications	40.1%	42.5%	0.56
	White^a^	Non-White	
Patients	847	297	
Patients on medications	339	126	
Proportion on medications	40.0%	42.4%	0.47
	Non-Hispanic^b^	Hispanic	
Patients	1044	110	
Patients on medications	425	43	
Proportion on medications	40.7%	39.1%	0.74

^a^Unknown race were omitted from comparison

^b^Unknown ethnicity were omitted from comparison

## Discussion

The COVID-19 pandemic presented challenges for chronic disease management across medical specialties. This study investigated whether the pandemic had significantly impacted initiation of pharmacologic treatment among patients newly diagnosed with osteoporosis. We found no significant differences in the 3-month and 6-month pharmacologic treatment rates between the pre-pandemic and pandemic cohorts. In addition, the time from osteoporosis diagnosis to therapy initiation and the prescribers were similar between the two cohorts, suggesting that the COVID-19 pandemic did not affect treatment rate or time to treatment.

Our findings contrasted with previously published literature on the impact of the pandemic on osteoporosis care. In particular, Cromer et al. reported the rates of DXA screening for osteoporosis declined during the pandemic [18]. In addition, many centers including theirs temporarily transitioned to virtual care, especially during the first wave of the pandemic. This led them to suggest that osteoporosis treatment was also impacted [18]. Fuggle et al. conducted a survey study of osteoporosis healthcare professionals and similarly found a rise in telemedicine appointments, delay in DXA scanning, and limited availability of medications for osteoporosis treatment [15]. Similar observations were made in the Netherlands [19]. However, the latter two studies were survey studies of healthcare professionals, whereas our study quantitatively measured treatment rate and time to treatment between two cohorts (pre-pandemic vs pandemic) that were similar in age, sex, and ethnicity. Our study also only looked at patients who were newly diagnosed.

Previous research has demonstrated osteoporosis is notoriously under screened and undertreated in males. In a single-center observational study, 94.5% of total screened subjects were female, while only 5.4% were male. In addition to drastically lower screening rate, when compared with female subjects the male subjects in this study were found to have an increased prevalence of fractures (50% in males vs. 31% in females) and secondary osteoporosis (66.67% in male vs. 20.83%% in females) [20]. This study clearly demonstrates the need for increased screening in male patients. A previous retrospective cohort study found that screening and treatment for osteoporosis following a hip fracture were low for both sexes, but lower for males (8%) compared to females (23.3%) [21, 22]. In another study evaluating osteoporosis following a distal radial fracture in patients 50 years and older, the male sex was found to be an independent predictor of failure to undergo screening and to receive proper treatment for osteoporosis [23]. study found no differences in treatment rates between male and female patients, but did identify an overall low treatment rate for osteoporosis in general (<45%).

With regard to racial differences in treatment, the literature has shown undertreatment in certain groups. A study from North Carolina found that white women had 5.96 times the odds of receiving a DEXA scan in the past and 2.97 times the odds of receiving guidance from a physician regarding osteoporosis compared to black women. Furthermore, this study reported white women were more likely to receive osteoporosis treatment from a physician compared to black women [24]. Another study at Kaiser Permanente of Southern California found that both white men and women were more likely to receive treatment before suffering a hip fracture, in comparison to African Americans [25]. Our study found similar osteoporosis treatment rates in White versus non-White patients as well as Hispanic versus non-Hispanic patients. Among all patients in this study, similar to when stratified for gender, there was an overall low rate of treatment (<45%).

It is important to note our study represented a single institution experience and might not be generalizable to other healthcare systems. Our institution is a tertiary referral center and had adequate resources and infrastructure to cope with the challenges in osteoporosis care brought about by the pandemic, whereas other under-resourced hospitals might have not been able to. At the peak of the first wave of the COVID-19 pandemic (March and April of 2020), our institution temporarily transitioned to telehealth for less than six weeks and quickly returned to in-person clinic appointments. We speculate there were no significant differences in treatment rate and time to treatment at our institution because most pharmacologic therapies for osteoporosis can be taken per os and prescribed electronically for patients to pick up at their pharmacy. Other than certain parenteral therapies such as denosumab (subcutaneous injection every six months) and zoledronate (intravenous administration yearly) that require medical supervision for administration, osteoporosis could be medically managed remotely through telehealth and telemedicine [26]. In fact, switching from parenteral to oral formulations has been recommended to improve osteoporosis care that would otherwise be compromised due to COVID-19 related restrictions [15, 27, 28].

Importantly, our study again highlighted the undertreatment of patients with osteoporosis, as only 40.5% of all patients newly diagnosed with osteoporosis were started on pharmacologic therapy within 6 months of diagnosis. This osteoporosis crisis is a major public health problem not only in the United States but also across the world [8, 29–31]. Despite guidelines from professional organizations such as the United States Preventive Services Taskforce (USPSTF) and National Osteoporosis Foundation (NOF) recommending screening for and treatment of osteoporosis in at-risk individuals, osteoporosis remains considerably underdiagnosed and undertreated [32, 33]. Building awareness and a team-based, multidisciplinary approach are essential to bridging the osteoporosis care gap that we are currently facing [34, 35].

Our study has important limitations, most notably its retrospective nature and single institution data, thus limiting generalizability. Our study only evaluated initiation but not maintenance of pharmacologic therapy for osteoporosis, which itself presents another challenge to osteoporosis management. Furthermore, our study could not determine why patients were not started on medical therapy, as there could have been factors other than COVID-19 that influenced treatment initiation after diagnosis. Lastly, our analyses did not subcategorize the pandemic cohort into two groups (vaccinated versus unvaccinated) based on when the COVID-19 vaccine became available at our institution. Vaccination status might have impacted access to osteoporosis diagnosis and treatment, which could not directly be addressed in our study.

## Conclusions

This is the first study to compare the impact of the COVID-19 pandemic on the pharmacologic treatment of patients who were newly diagnosed with osteoporosis. In our retrospective comparative study, we found only 40.5% of patients with newly diagnosed osteoporosis were treated pharmacologically within six months of diagnosis, and the COVID-19 pandemic did not significantly affect treatment rates. Bisphosphonates were the most often prescribed medication group. Further studies are needed to better understand patient-, provider-, and system-specific factors contributing to the low treatment rates of patients newly diagnosed with osteoporosis.

## Supporting information

S1 Data (XLSX)
